# Correlates of healthcare-seeking behavior for acute gastroenteritis—United States, October 1, 2016 –September 30, 2017

**DOI:** 10.1371/journal.pone.0293739

**Published:** 2023-10-31

**Authors:** Benjamin D. Hallowell, Rachel M. Burke, S. Bianca Salas, Holly Groom, Judy L. Donald, Claire P. Mattison, Mark A. Schmidt, Aron J. Hall

**Affiliations:** 1 Division of Viral Diseases, Centers for Disease Control and Prevention, Atlanta, Georgia, United States of America; 2 Epidemic Intelligence Service, Centers for Disease Control and Prevention, Atlanta, Georgia, United States of America; 3 Center for Health Research, Kaiser Permanente Northwest, Portland, Oregon, United States of America; 4 Oak Ridge Institute for Science and Education, Oak Ridge, Tennessee, United States of America; LSHTM: London School of Hygiene & Tropical Medicine, UNITED KINGDOM

## Abstract

**Background:**

In the United States, public health surveillance systems often underestimate the burden of acute gastroenteritis (AGE) because they only identify disease among those who interact with the healthcare system.

**Objective:**

To identify factors associated with healthcare-seeking behavior among individuals experiencing community-acquired AGE.

**Methods:**

From October 2016 –September 2017, we conducted a weekly, age-stratified, random sample of Kaiser Permanente Northwest members located in northwest Oregon and southwest Washington, United States. Individuals who completed the online survey and experienced AGE were included in the analysis. Univariate and multivariable logistic regressions were performed to identify predictors of healthcare-seeking behavior.

**Results:**

Of the 3,894 survey respondents, 395 experienced an AGE episode and were eligible for analysis, of whom, 82 (21%) sought care for their AGE episode. In the final multivariable model, individuals with a concurrent fever (odds ratio [OR]: 4.76, 95% confidence interval [95% CI]: 2.48–9.13), increased diarrhea duration (≥6 days vs 1–4 days, OR: 4.22, 95% CI: 1.78–10.03), or increased vomiting duration (≥3 days vs 1 days, OR: 2.97, 95% CI: 1.22–7.26), were significantly more likely to seek healthcare. In the adjusted model, no sociodemographic or chronic disease variables were associated with healthcare-seeking behavior.

**Conclusion:**

These findings suggest that individuals with a short duration of AGE and those without concurrent fever are underrepresented in healthcare facility-based surveillance systems.

## Introduction

Most public health surveillance systems for acute gastroenteritis (AGE) are dependent on individuals with AGE seeking healthcare and, for assessing specific AGE pathogens, providing a stool specimen for diagnosis of their illness. However, most AGE episodes are mild and self-limiting, and as such, approximately 80% of individuals experiencing AGE do not seek care [1–4], and as few as 0.3% of individuals seek care and provide a stool specimen [5]. While many studies have identified factors associated with healthcare-seeking behavior among children in developing countries [6–10], few have assessed factors associated with healthcare-seeking behavior in developed countries or across the full age spectrum [5, 11]. Understanding the factors that are associated with healthcare-seeking behavior can help surveillance systems more accurately estimate the burden of AGE and identify characteristics associated with underreporting. This information is essential to accurately interpret surveillance-derived data, which in turn helps guide public health prevention and resource allocation efforts.

## Methods

To identify correlates associated with healthcare-seeking behavior we utilized data collected from the Community Acute Gastroenteritis (CAGE) Study conducted within an integrated healthcare delivery system of over 600,000 members in northwest Oregon and southwest Washington, United States [12]. The study methodology has been previously described [13]. In brief, from October 1, 2016 to September 30, 2017, 52 weekly, age-stratified, random samples of Kaiser Permanente Northwest (KPNW) members were recruited to complete an online survey pertaining to community acquired AGE. The survey collected sociodemographic information, the frequency of diarrhea and/or vomiting in the preceding 30 days, and the presence of any chronic gastroenteritis conditions. Among individuals reporting diarrhea and/or vomiting, information on symptoms, related healthcare-seeking behaviors, and treatments received were recorded. Separate surveys were used for minors (<18 years) and adults. For all minors <18 years a legal guardians provided informed consent and provided responses to the survey on their behalf. Information on insurance status and other pre-existing chronic diseases present at the time of survey completion were obtained from electronic health records.

Individuals were included in the analysis if they completed the online survey and reported an AGE episode, defined as three or more loose stools in any 24-hour period or any vomiting, in the last 30 days. Individuals with a self-reported chronic gastroenteritis condition who did not report any vomiting in the last 30 days were excluded from all analyses. A chronic gastroenteritis condition was defined as an illness lasting longer than 1 month in which diarrhea or vomiting is a major symptom, including irritable bowel syndrome, ulcerative colitis, and Crohn’s disease. Healthcare-seeking behavior was defined as any reported contact with a health professional related to their AGE episode, including the following: call to nurse phone line, email to healthcare provider, video consultation with healthcare provider, in-person clinic visit, urgent care visit, emergency room visit, and admission to the hospital.

Differences in sociodemographic, clinical, and chronic disease factors among individuals who sought care and individuals who did not seek care were compared through chi-square tests. Age at the time of survey completion was categorized (<5, 5–17, 18–44, 45–64, ≥65 years). Prior to analysis, duration of diarrhea, maximum episodes of diarrhea in 24 hours, duration of vomiting, and maximum episodes of vomiting in 24 hours were recoded categorically to align with the modified Vesikari scoring system [14]. In addition, race, insurance status, and age variables were collapsed to allow for adequate sample size for comparisons among groups. All other variables are presented as recorded in the survey. All variables with >10% of data missing were excluded from both bivariate and multivariable logistic regressions.

The final multivariable model was built using a forward selection approach. Independent variables were added to the model individually based on their ability to significantly improve model fit using Akaike Information Criterion values. All variables with a *p-*value <0.2 in univariate analyses were eligible for inclusion. Age and gender were identified as potential confounding variables *a priori* and were included in the model. Crude odds ratios (OR), multivariate adjusted odds ratios (aOR), and 95% confidence intervals (95% CI) were calculated using SAS v9.4 (SAS Institute; Cary, NC).

This project was reviewed and approved by the KPNW Institutional Review Board (FWA00002344). Participants provided informed consent to participate in this study.

## Results

In total, 3,894 individuals participated in the CAGE study. After excluding respondents who did not experience AGE in the last 30 days (n = 3,422) and those with a chronic gastrointestinal condition who did not report any vomiting in the last 30 days (n = 77), 395 were eligible for inclusion in the analysis.

**Fig 1:**
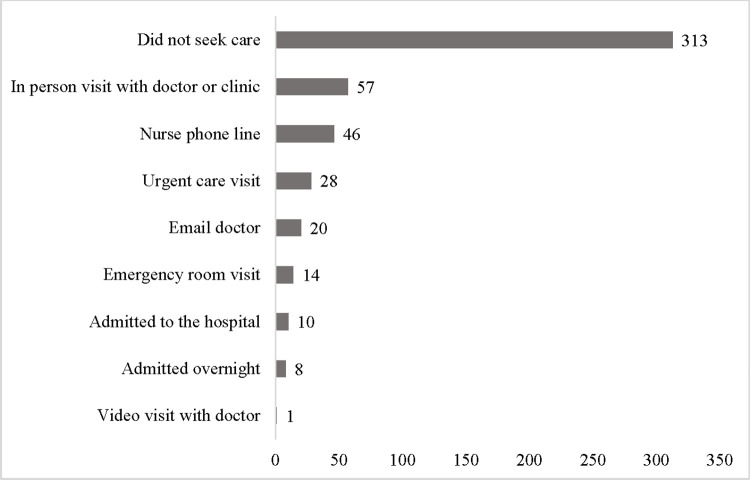
Self-reported ways individuals experiencing acute gastroenteritis sought healthcare (multiple healthcare encounter types allowed; n = 395),—United States, October 1, 2016—September 30, 2017.

Of the 395 respondents with recent AGE, 82 (21%) sought care for their AGE episode. Among individuals who sought care, an in-person clinic visit (70%), calling the nurse phone line (56%) or visiting an urgent care clinic (34%) were the most common (Fig 1). Most respondents with an AGE episode that sought care had at least two different encounter types within the healthcare system, with 34%, 33%, 21%, and 13% reporting one, two, three, or four or more different healthcare encounter types, respectively, for their AGE episode. Most individuals (45%) had remote (email, video call, phone call) and in-person (visit with a doctor or clinic, emergency room visit, admission to hospital) care, while 21% received only remote care, and 34% only received in-person care. Individuals reported seeking care because their symptoms were bad (90%), their symptoms lasted a long time (79%), they wanted to know what made them ill (76%), or their home remedies did not make them feel better (64%; Table 1).

**Table 1:** Self-reported reasons individuals experiencing acute gastroenteritis sought healthcare (n = 82)—United States, October 1, 2016—September 30, 2017*.

Reason	Frequency n (%)
Because my symptoms were so bad	
Yes	63 (90)
No	7 (10)
Because my symptoms lasted a long time	
Yes	52 (79)
No	14 (21)
I didn’t want to infect other people	
Yes	28 (47)
No	31 (53)
I needed a doctor’s approval to go back to work	
Yes	9 (17)
No	44 (83)
I wanted to know what made me ill	
Yes	52 (76)
No	16 (24)
My home remedies did not make me feel better	
Yes	42 (64)
No	24 (36)
I saw a news story encouraging people with stomach illness to go to the doctor	
Yes	3 (6)
No	49 (94)
My friends, family, and coworkers encouraged me to go to the doctor	
Yes	26 (47)
No	29 (53)
I just got back from a trip where I may have gotten my stomach illness	
Yes	3 (6)
No	49 (94)

*Values in columns may not sum to the total because of missing values.

When comparing demographic factors in bivariate analyses, individuals aged 45–65 years were less likely to seek care (OR: 0.35, 95% CI: 0.17, 0.74) when compared to individuals age <5. No other demographic variables, including sex, were significantly associated with healthcare utilization (Table 2). Symptomatically, individuals who sought care were more likely to have a longer diarrhea duration (5 days vs 1–4 days, OR: 2.47, 95% CI: 1.20–5.08; ≥6 days vs 1–4 days, OR: 3.55, 95% CI: 1.67–7.55), have vomiting (OR: 3.89; 95% CI: 2.22–6.70), vomit more times in any 24-hour period (1 time vs 0 times, OR: 3.74; 95% CI: 1.77–7.90; 2–4 times vs 0 times, OR: 3.67; 95% CI; 1.94–6.96; ≥5 times vs 0 times, OR: 4.19; 95% CI; 1.97–8.93), have a longer vomiting duration (≥3 days vs 1 day, OR: 3.52; 95% CI:1.62–7.65), and have additional concurrent symptoms including sore throat (OR: 2.55; 95% CI: 1.45–4.48), cough (OR: 2.52; 95% CI: 1.49–4.26), or fever (OR: 5.38; 95% CI: 3.15–9.16). When looking at the associations between pre-existing chronic conditions and healthcare seeking behavior, individuals with lung disease were more likely to seek care for AGE (OR: 5.30, 95% CI: 2.02–13.89; Table 2).

**Table 2:** Distribution and bivariate associations between demographic characteristics, clinical factors, and chronic disease history by healthcare seeking behavior among individuals with self-reported AGE (n = 395)—United States, October 1, 2016—September 30, 2017*.

Characteristic	Individuals experiencing AGE who sought healthcare n = 82	Individuals experiencing AGE who did not seek healthcare n = 313	Bivariate Odds Ratio (95% CI)	p-value
**Demographic**				
**Age (years)**				
<5	20 (24.4)	45 (14.4)	Ref	0.069
5–17	10 (12.2)	26 (8.3)	0.87 (0.35, 2.13)	
18–44	24 (29.3)	96 (30.7)	0.56 (0.28, 1.12)	
45–64	16 (19.5)	103 (32.9)	0.35 (0.17, 0.74)	
≥65	12 (14.6)	43 (13.7)	0.63 (0.27, 1.44)	
**Sex**				
Male	35 (42.7)	118 (37.7)	Ref	0.410
Female	47 (57.3)	195 (62.3)	0.81 (0.50, 1.33)	
**Sexual Preference** ^**4**^				
Heterosexual	45 (88.2)	218 (92.0)	-	
Lesbian	0 (0.0)	3 (1.3)	-	
Gay	1 (2.0)	2 (0.8)	-	
Bisexual	4 (7.8)	9 (3.8)	-	
Other	1 (2.0)	5 (2.1)	-	
**Race**				
White	68 (82.9)	269 (85.9)	Ref	0.302
Other	14 (17.1)	39 (12.7)	1.42 (0.73, 2.77)	
**Ethnicity**				
Non-Hispanic	64 (85.3)	256 (90.8)	Ref	0.173
Hispanic	11 (14.7)	26 (9.2)	1.69 (0.79, 3.61)	
**Insurance Status**				
Commercial Only	62 (75.6)	250 (79.9)	Ref	0.400
Medicare, Medicaid, or None	20 (24.4)	63 (20.1)	1.28 (0.72, 2.27)	
**Household Income** ^**4**^				
≤ $50,000	14 (37.8)	33 (25.2)	-	
$50,000-$75,000	6 (16.2)	27 (20.6)	-	
$75,000-$100,000	7 (18.9)	27 (20.6)	-	
$100,000 ≤	10 (27.0)	44 (33.6)	-	
**Education** ^**1**^ ^,^ ^**4**^				
Less than high school	1 (2.0)	3 (1.3)	-	
High school or equivalent	6 (11.8)	23 (9.7)	-	
Some college	18 (35.3)	90 (37.8)	-	
College graduate	20 (39.2)	79 (33.2)	-	
Post graduate	6 (11.8)	43 (18.1)	-	
**Occupation** ^**1**^ ^,^ ^**4**^				
Health care				
No	40 (78.4)	192 (81.7)	-	
Yes	11 (21.6)	43 (18.3)	-	
Food Service				
No	49 (94.2)	228 (95.8)	-	
Yes	3 (5.8)	10 (4.2)	-	
Education				
No	48 (92.3)	203 (85.7)	-	
Yes	4 (7.7)	34 (14.3)	-	
**Residence Setting**				
City or urban area	37 (45.7)	136 (44.0)	Ref	0.992
Suburban area	31 (38.3)	123 (39.8)	0.93 (0.54, 1.58)	
Town or village	4 (4.9)	18 (5.8)	0.82 (0.26, 2.56)	
Rural but not on a farm	7 (8.6)	26 (8.4)	0.99 (0.40, 2.46)	
On a farm	2 (2.5)	6 (1.9)	1.23 (0.24, 6.32)	
**Did you travel outside of the US in the previous 60 days?**				
No	80 (97.6)	300 (96.5)	Ref	0.623
Yes	2 (2.4)	11 (3.5)	0.68	
**Pregnant at any time in the past 30 days** ^**2**^ ^,^ ^**4**^				
No	45 (95.7)	193 (99.0)	-	
Yes	2 (4.3)	2 (1.0)	-	
**Contact with individual with diarrhea or vomiting in the past 30 days** ^**4**^				
No	37 (53.6)	125 (48.6)	-	
Yes	32 (46.7)	132 (51.4)	-	
**Contact with a household member with diarrhea or vomiting in the past 30 days** ^**4**^				
No	50 (72.5)	156 (60.7)	-	
Yes	19 (27.5)	101 (39.3)	-	
**Employed** ^**3**^ ^,^ ^**4**^				
No	13 (31.0)	49 (32.0)	-	
Yes	29 (69.0)	104 (68.0)	-	
**Missed work due to illness in last 30 days** ^**3**^ ^,^ ^**4**^				
No	1 (7.1)	55 (68.7)	-	
Yes	13 (92.9)	25 (31.3)	-	
**How many paid hours of work did you miss?** ^**3**^ ^,^ ^**4**^				
Median (IQR)	16 (5.5, 40: n = 12)	8 (4, 14; n = 25)	-	
**How many unpaid hours of work did you miss?** ^**3**^ ^,^ ^**4**^				
Median (IQR)	0 (0, 24: n = 11)	0 (0, 5; n = 22)	-	
**In the past 30 days, did this illness keep you from doing other, usual activities** ^**3**^ ^,^ ^**4**^				
No	2 (8.0)	52 (41.6)	-	
Yes	23 (92.0)	73 (58.4)	-	
**Did you require assistance from others over the last 30 days due to your illness?** ^**3**^ ^,^ ^**4**^				
No	20 (47.6)	135 (86.5)	-	
Yes	22 (52.4)	21 (13.5)	-	
**Clinical**				
**Diarrhea in the past 30 days?**				
No (vomiting only)	18 (22.2)	48 (15.4)	Ref	0.148
Yes (with or without vomiting)	63 (77.8)	263 (84.6)	0.64 (0.35, 1.17)	
**Were any stools bloody?**				
No	71 (92.2)	284 (96.0)	Ref	0.180
Yes	6 (7.8)	12 (4.0)	2.00 (0.73, 5.51)	
**Maximum stools in 24-hour period**				
0 (vomiting only)	18 (22.2)	48 (15.7)	Ref	0.526
1–3	21 (25.9)	90 (29.5)	0.62 (0.30, 1.28)	
4–5	25 (30.9)	107 (35.1)	0.62 (0.31, 1.25)	
≥6	17 (21.0)	60 (19.7)	0.76 (0.35, 1.62)	
**Diarrhea Duration (days)**				
0 (vomiting only)	18 (22.2)	48 (15.5)	2.19 (1.14, 4.19)	0.002
1–4	35 (43.2)	204 (66.3)	Ref	
5	14 (17.3)	33 (10.7)	2.47 (1.20, 5.08)	
≥6	14 (17.3)	23 (7.5)	3.55 (1.67, 7.55)	
**Vomiting in the last 30 days?**				
No (diarrhea only)	20 (24.4)	173 (55.4)	Ref	<0.001
Yes (with or without diarrhea)	62 (75.6)	139 (44.6)	3.86 (2.22, 6.70)	
**Maximum times vomited in any 24-hour period**				
0 (diarrhea only)	20 (25.0)	173 (56.0)	Ref	<0.001
1	16 (20.0)	37 (12.0)	3.74 (1.77, 7.90)	
2–4	28 (35.0)	66 (21.4)	3.67 (1.94, 6.96)	
≥5	16 (20.0)	33 (10.7)	4.19 (1.97, 8.93)	
**Vomiting Duration (days)**				
0 (diarrhea only)	20 (24.4)	173 (55.6)	0.41 (0.21, 0.77)	<0.001
1	25 (30.5)	88 (28.3)	Ref	
2	18 (22.0)	31 (10.0)	2.04 (0.98, 4.25)	
≥3	19 (23.2)	19 (6.1)	3.52 (1.62, 7.65)	
**Concurrent Symptoms**				
Sore throat				
No	50 (65.8)	245 (83.0)	Ref	0.001
Yes	26 (34.2)	50 (17.0)	2.55 (1.45, 4.48)	
Cough				
No	49 (60.5)	243 (79.4)	Ref	0.001
Yes	32 (39.5)	63 (20.6)	2.52 (1.49, 4.26)	
Fever				
No	39 (47.6)	234 (83.0)	Ref	<0.001
Yes	43 (52.4)	48 (17.0)	5.38 (3.15, 9.16)	
**Chronic Conditions**				
**Cancer**				
No	80 (97.6)	296 (94.6)	Ref	0.273
Yes	2 (2.4)	17 (5.4)	0.44 (0.10, 1.92)	
**Diabetes**				
No	77 (93.9)	282 (90.1)	Ref	0.291
Yes	5 (6.1)	31 (9.9)	0.59 (0.22, 1.57)	
**Heart Disease**				
No	74 (90.2)	291 (93.0)	Ref	0.408
Yes	8 (9.8)	22 (7.0)	1.43 (0.61, 3.34)	
**Liver Disease**				
No	81 (98.8)	309 (98.7)	Ref	0.967
Yes	1 (1.2)	4 (1.3)	0.95 (0.11, 8.65)	
**Kidney**				
No	76 (92.7)	300 (95.8)	Ref	0.240
Yes	6 (7.3)	13 (4.1)	1.82 (0.67, 4.95)	
**Lung Disease**				
No	72 (87.8)	305 (97.4)	Ref	0.001
Yes	10 (12.2)	8 (2.6)	5.30 (2.02, 13.89)	
**Metabolic Disease**				
No	69 (84.1)	248 (79.3)	Ref	0.322
Yes	13 (15.9)	65 (20.8)	0.72 (0.37, 1.38)	

*Values in columns may not sum to the total because of missing values.

^1^Restricted to individuals ≥18 years old.

^2^Restricted to women.

^3^Restricted to individuals who took the survey after April 7, 2017 when the questions were added (n = 200).

^4^Not included in bivariate analyses due to >10% of data missing. OR: Odds Ratio. CI: Confidence Interval.

In the final multivariable model, diarrhea duration, vomiting duration, and fever were the only significant predictors of healthcare-seeking behavior among individuals experiencing AGE, with no sociodemographic or chronic disease variables significantly associated (Table 3). As diarrhea duration increased (≥6 days vs 1–4 days, aOR: 4.22, 95% CI: 1.78–10.03) or vomiting duration increased (≥3 days vs 1 day, aOR: 2.97, 95% CI: 1.22–7.26) individuals were more likely to seek care, while those not experiencing vomiting (0 days vs 1 day, aOR: 0.46, 95% CI: 0.21–0.99) were less likely to seek care. Individuals who had a concurrent fever with their diarrhea and/or vomiting were also more likely (OR: 4.76, 95% CI: 2.48–9.13) to seek healthcare.

**Table 3:** Multivariable model predicting healthcare seeking behavior among individuals with self-reported AGE (n = 360)—United States, October 1, 2016—September 30, 2017*.

Characteristic	Bivariate OR (95% CI)	p-value	Multivariate aOR (95% CI)	p-value
**Age (years)**				
<5	Ref	0.069	Ref	0.531
5–17	0.87 (0.35, 2.13)		0.70 (0.25, 2.01)	
18–44	0.56 (0.28, 1.12)		0.98 (0.41, 2.34)	
45–64	0.35 (0.17, 0.74)		0.62 (0.25, 1.52)	
≥65	0.63 (0.27, 1.44)		1.37 (0.49, 3.84)	
**Sex**				
Male	Ref	0.410	Ref	0.474
Female	0.81 (0.50, 1.33)		0.80 (0.43, 1.49)	
**Diarrhea Duration (days)**				
0	2.19 (1.14, 4.19)	0.002	1.16 (0.52, 2.60)	0.010
1–4	Ref		Ref	
5	2.47 (1.20, 5.08)		1.86 (0.77, 4.49)	
≥6	3.55 (1.67, 7.55)		4.22 (1.78, 10.03)	
**Vomiting Duration (days)**				
0	0.41 (0.21, 0.77)	<0.001	0.46 (0.21, 0.99)	0.002
1	Ref		Ref	
2	2.04 (0.98, 4.25)		1.06 (0.44, 2.47)	
≥3	3.52 (1.62, 7.65)		2.97 (1.22, 7.26)	
**Concurrent Symptoms**				
Fever				
No	Ref	<0.001	Ref	<0.001
Yes	5.38 (3.15, 9.16)		4.76 (2.48, 9.13)	

*Restricted to variables with <10% of data missing. OR: Odds Ratio. aOR: Adjusted Odds Ratio. CI: Confidence Interval.

## Discussion

Similar to other work in developed countries (range: 19–36.5%), in this study, one out of every five individuals experiencing AGE sought care, with most having an in-person visit with a healthcare provider [1–3, 5]. Individuals were more likely to seek care as the duration of their diarrhea or vomiting increased, and if a fever accompanied their AGE symptoms. These findings align with individual’s self-reported reasons for seeking care, which included that their symptoms were bad, lasted a long time, or that their home remedies did not make them feel better. In this study, no sociodemographic characteristics were significantly associated with healthcare-seeking behavior in our final multivariable model.

These findings support other work that has found that a long duration of illness [5, 11], concurrent fever [2, 5, 15], longer diarrhea duration [2], and vomiting [2, 16] were significantly associated with care seeking behavior in multivariable analyses. Other work has additionally shown other concurrent symptoms, including headaches [11] and abdominal cramps [15] to be associated with healthcare-seeking behavior, but these variables were not assessed in our study. While other studies have found that individuals with lower household incomes were more likely to seek care [2], 57% of our sample omitted responses to this variable and as such, household income could not be assessed as a potential healthcare-seeking factor in this study. While not significant in the bivariate or multivariable models, there was a higher proportion of males who sought care when compared to females, similar to previous work exploring healthcare seeking behavior for AGE [2, 5].

In prior work, age is often associated with healthcare-seeking behavior with children (<5 years or <15 years) and older adults (65+ years) more likely to seek care when compared to other age groups [2, 3, 11, 15, 17]. While age was not found to be a significant predictor in our final multivariable model, similar patterns were observed. Of note, when restricting the analysis to individuals experiencing diarrhea (with or without vomiting), children were significantly more likely to have healthcare-seeking behavior when compared to older adults (45–64 years). However, when limiting the analysis to individuals experiencing vomiting (with or without diarrhea) no association with age was observed in bivariate or multivariable models. Sample size prevented us from stratifying the analyses by age group.

In the healthcare setting, AGE treatment is often symptom-based, with little emphasis placed on finding the underlying cause of disease. However, 76% of individuals who sought care in this study indicated they did so in part to identify what made them ill. This desire on behalf of the patient may encourage the use of multi-pathogen detection assays, which could improve the patient’s healthcare experience, reduce the inappropriate prescription of antibiotics, and enhance AGE surveillance efforts.

This work provides evidence that AGE surveillance systems are more likely to detect cases that have a longer duration of diarrhea or vomiting, or additional concurrent symptoms (e.g. fever). However, while these individuals may be more likely to interact with the healthcare system, as only 11 individuals who interacted with the healthcare system were asked to provide a stool sample (9 of which provided a sample), we could not assess if these factors were also associated with diagnostic testing (i.e. submitting a stool sample).

The strengths of this study are that it was a random, age-stratified, sample of KPNW members. Similar to all studies of this nature, the results are subject to recall bias. With regards to limitations, it is possible that additional characteristics are associated with healthcare-seeking behavior but did not have sufficient sample sizes to be detected in this study and the limited sample size prevented stratification in the multivariate model. Additionally, information on several key predictor variables (e.g. income) were not reported by enough participants and were omitted from bivariate and multivariate models. Finally, this population was composed of KPNW members living in the United States, who are nearly all insured, as such these findings may not apply to uninsured populations or populations in other countries.

## Conclusion

AGE surveillance systems based on interactions with the healthcare system likely underestimate the true burden of AGE in the population as only one in five individuals seek care for their AGE episode. AGE cases that seek healthcare are more likely to have had a longer duration of diarrhea, a longer duration of vomiting, and the presence of other concurrent symptoms (i.e., fever) when compared to individuals who are not detected. These findings can help guide further refinement and appropriate interpretation of AGE surveillance data, which in turn can be used to develop targeted interventions to prevent AGE.

## Supporting information

S1 Checklist STROBE statement—checklist of items that should be included in reports of observational studies. (DOCX)
