# Supplementary material for: Correlates of healthcare-seeking behavior for acute gastroenteritis—United States, October 1, 2016 –September 30, 2017
Source: PLoS One. 2023 Oct 31;18(10):e0293739. doi: 10.1371/journal.pone.0293739 (PMC10617691; doi:10.1371/journal.pone.0293739)
Supplement: S1 Checklist — (DOCX) [file pone.0293739.s001.docx]

STROBE Statement—checklist of items that should be included in reports of observational studies

**Correlates of healthcare-seeking behavior for acute gastroenteritis**

Hallowell et al. 2023

|  | | Item No. | | Recommendation | Page  No. | | | Relevant text from manuscript | |
| --- | --- | --- | --- | --- | --- | --- | --- | --- | --- |
| **Title and abstract** | | 1 | | (*a*) Indicate the study’s design with a commonly used term in the title or the abstract | 2 | | | “From October 2016 – September 2017 we conducted a weekly, age-stratified, random sample of Kaiser Permanente Northwest members. Individuals who completed the online survey and experienced AGE were included in the analysis.” | |
|  |  |  |  | (*b*) Provide in the abstract an informative and balanced summary of what was done and what was found | 2 | | | “Univariate and multivariable logistic regressions were performed to identify predictors of healthcare-seeking behavior. Of the 3,894 survey respondents, 395 experienced an AGE episode and were eligible for analysis, of whom, 82 (21%) sought care for their AGE episode. In the final multivariable model, individuals with a concurrent fever (odds ratio [OR]: 4.76, 95% confidence interval [95% CI]: 2.48-9.13), increased diarrhea duration (≥6 days vs 1-4 days, OR: 4.22, 95% CI: 1.78-10.03), or increased vomiting duration (≥3 days vs 1 days, OR: 2.97, 95% CI: 1.22-7.26), were significantly more likely to seek healthcare. “ | |
| Introduction | | | | | | |  | |  |
| Background/rationale | | 2 | | Explain the scientific background and rationale for the investigation being reported | 3 | | | “While many studies have identified factors associated with healthcare-seeking behavior among children in developing countries,^4-8^ few have assessed factors associated with healthcare-seeking behavior in developed countries or across the full age spectrum.” | |
| Objectives | | 3 | | State specific objectives, including any prespecified hypotheses | 3 | | | “Understanding the factors that are associated with healthcare-seeking behavior can help surveillance systems more accurately estimate the burden of AGE and identify characteristics associated with underreporting.” | |
| Methods | | | | | | |  | |  |
| Study design | | 4 | | Present key elements of study design early in the paper | 3 | | | “In brief, beginning in October 2016, 52 weekly, age-stratified, random samples of Kaiser Permanente Northwest (KPNW) members were recruited to complete an online survey pertaining to community acquired AGE. “ | |
| Setting | | 5 | | Describe the setting, locations, and relevant dates, including periods of recruitment, exposure, follow-up, and data collection | 3 | | | “In brief, beginning in October 2016, 52 weekly, age-stratified, random samples of Kaiser Permanente Northwest (KPNW) members were recruited to complete an online survey pertaining to community acquired AGE. “ | |
| Participants | | 6 | | (*a*) *Cohort study*—Give the eligibility criteria, and the sources and methods of selection of participants. Describe methods of follow-up  *Case-control study*—Give the eligibility criteria, and the sources and methods of case ascertainment and control selection. Give the rationale for the choice of cases and controls  *Cross-sectional study*—Give the eligibility criteria, and the sources and methods of selection of participants | 4 | | | “Individuals were included in the analysis if they completed the online survey and reported an AGE episode, defined as three or more loose stools in any 24-hour period or any vomiting, in the last 30 days. Individuals with a self-reported chronic gastroenteritis condition who did not report any vomiting in the last 30 days were excluded from all analyses. “ | |
|  |  |  |  | (*b*) *Cohort study*—For matched studies, give matching criteria and number of exposed and unexposed  *Case-control study*—For matched studies, give matching criteria and the number of controls per case |  | | | n/a | |
| Variables | | 7 | | Clearly define all outcomes, exposures, predictors, potential confounders, and effect modifiers. Give diagnostic criteria, if applicable | 4-5 | | | AGE episode, defined as three or more loose stools in any 24-hour period or any vomiting, in the last 30 days. Healthcare-seeking behavior was defined as any reported contact with a health professional related to their AGE episode.  Age at the time of survey completion was categorized (<5, 5–¬17, 18–44, 45–64, ≥65 years). Prior to analysis, duration of diarrhea, maximum episodes of diarrhea in 24 hours, duration of vomiting, and maximum episodes of vomiting in 24 hours were recoded categorically to align with the modified Vesikari scoring system. In addition, race, insurance status, and age variables were collapsed to allow for adequate sample size for comparisons among groups. | |
| Data sources/ measurement | | 8* | | For each variable of interest, give sources of data and details of methods of assessment (measurement). Describe comparability of assessment methods if there is more than one group | 3-4 | | | The survey collected sociodemographic information, the frequency of diarrhea and/or vomiting in the preceding 30 days, and the presence of any chronic gastroenteritis conditions. Among individuals reporting diarrhea and/or vomiting, information on symptoms, related healthcare-seeking behaviors, and treatments received were recorded. Separate surveys were used for minors (<18 years) and adults. For all minors <18 years a legal guardian provided responses to the survey on their behalf. Information on insurance status and other pre-existing chronic diseases present at the time of survey completion were obtained from electronic health records. | |
| Bias | | 9 | | Describe any efforts to address potential sources of bias | 4-5 | | | Age and gender were identified as potential confounding variables *a priori* and were forced into the model. | |
| Study size | | 10 | | Explain how the study size was arrived at | 3 | | | The study methodology has been previously described.^10^ | |
|  |  | |  | |  |  | | | |
| Quantitative variables | 11 | | Explain how quantitative variables were handled in the analyses. If applicable, describe which groupings were chosen and why | | 4 | Prior to analysis, duration of diarrhea, maximum episodes of diarrhea in 24 hours, duration of vomiting, and maximum episodes of vomiting in 24 hours were recoded categorically to align with the modified Vesikari scoring system.^11^ In addition, race, insurance status, and age variables were collapsed to allow for adequate sample size for comparisons among groups. All other variables are presented as recorded in the survey. | | | |
| Statistical methods | 12 | | (*a*) Describe all statistical methods, including those used to control for confounding | |  | Differences in sociodemographic, clinical, and chronic disease factors among individuals who sought care and individuals who did not seek care were compared through chi-squared tests. The final multivariable model was built using a forward selection approach. Independent variables were added to the model individually based on their ability to significantly improve model fit using Akaike Information Criterion values. All variables with a *p-*value <0.2 in univariate analyses were eligible for inclusion. Age and gender were identified as potential confounding variables *a priori* and were forced into the model. Bivariate odds ratios (OR), multivariate adjusted odds ratios (aOR), and 95% confidence intervals (95% CI) were calculated using SAS v9.4 | | | |
|  |  |  | (*b*) Describe any methods used to examine subgroups and interactions | |  |  | | | |
|  |  |  | (*c*) Explain how missing data were addressed | | 4, 7-10 | “All variables with >10% of data missing were excluded from both bivariate and multivariable logistic regressions.”, Table 2 | | | |
|  |  |  | (*d*) *Cohort study*—If applicable, explain how loss to follow-up was addressed  *Case-control study*—If applicable, explain how matching of cases and controls was addressed  *Cross-sectional study*—If applicable, describe analytical methods taking account of sampling strategy | | 5 | Age and gender were identified as potential confounding variables *a priori* and were forced into the model. | | | |
|  |  |  | (*e*) Describe any sensitivity analyses | | n/a |  | | | |
| Results | | | | | | | | |  |
| Participants | 13* | | (a) Report numbers of individuals at each stage of study—eg numbers potentially eligible, examined for eligibility, confirmed eligible, included in the study, completing follow-up, and analysed | | 5 | “In total, 3,894 individuals participated in the CAGE study. After excluding respondents who did not experience AGE in the last 30 days (n=3,422) and those with a chronic gastrointestinal condition who did not report any vomiting in the last 30 days (n=77), 395 were eligible for inclusion in the analysis. “ | | | |
|  |  |  | (b) Give reasons for non-participation at each stage | | 5 | See above | | | |
|  |  |  | (c) Consider use of a flow diagram | |  | n/a | | | |
| Descriptive data | 14* | | (a) Give characteristics of study participants (eg demographic, clinical, social) and information on exposures and potential confounders | | 7-10 | Table 2 | | | |
|  |  |  | (b) Indicate number of participants with missing data for each variable of interest | | 4, 7-10 | “All variables with >10% of data missing were excluded from both bivariate and multivariable logistic regressions.”, Table 2 | | | |
|  |  |  | (c) *Cohort study*—Summarise follow-up time (eg, average and total amount) | | n/a |  | | | |
| Outcome data | 15* | | *Cohort study*—Report numbers of outcome events or summary measures over time | | n/a |  | | | |
|  |  |  | *Case-control study—*Report numbers in each exposure category, or summary measures of exposure | | n/a |  | | | |
|  |  |  | *Cross-sectional study—*Report numbers of outcome events or summary measures | | 5 | Of the 395 respondents with recent AGE, 82 (21%) sought care for their AGE episode. | | | |
| Main results | 16 | | (*a*) Give unadjusted estimates and, if applicable, confounder-adjusted estimates and their precision (eg, 95% confidence interval). Make clear which confounders were adjusted for and why they were included | | 7-11 | Table 2, table 3 | | | |
|  |  |  | (*b*) Report category boundaries when continuous variables were categorized | | 7-11 | Table 2, table 3 | | | |
|  |  |  | (*c*) If relevant, consider translating estimates of relative risk into absolute risk for a meaningful time period | |  | n/a | | | |

Continued on next page

| Other analyses | 17 | Report other analyses done—eg analyses of subgroups and interactions, and sensitivity analyses |  | n/a |
| --- | --- | --- | --- | --- |
| Discussion | | | | |
| Key results | 18 | Summarise key results with reference to study objectives | 11 | ‘Individuals were more likely to seek care as the duration of their diarrhea or vomiting increased, and if a fever accompanied their AGE symptoms” |
| Limitations | 19 | Discuss limitations of the study, taking into account sources of potential bias or imprecision. Discuss both direction and magnitude of any potential bias | 13 | “Similar to all studies of this nature, the results are subject to recall bias. With regards to limitations, it is possible that additional characteristics are associated with healthcare-seeking behavior but did not have sufficient sample sizes to be detected in this study. Additionally, information on several key predictor variables (e.g. income) were not reported by enough participants and were omitted from bivariate and multivariate models. ” |
| Interpretation | 20 | Give a cautious overall interpretation of results considering objectives, limitations, multiplicity of analyses, results from similar studies, and other relevant evidence | 13 | “AGE surveillance systems based on interactions with the healthcare system likely underestimate the true burden of AGE in the population as only one in five individuals seek care for their AGE episode. AGE cases that seek healthcare are more likely to have had a longer duration of diarrhea, a longer duration of vomiting, and the presence of other concurrent symptoms (i.e., fever) when compared to individuals who are not detected.” |
| Generalisability | 21 | Discuss the generalisability (external validity) of the study results | 13 | “Finally, this population was composed of KPNW members who are nearly all insured, as such these findings may not apply to uninsured populations.” |
| Other information | |  | | |
| Funding | 22 | Give the source of funding and the role of the funders for the present study and, if applicable, for the original study on which the present article is based | n/a | “This work was supported by the CDC Foundation (institutional research funding to the Kaiser Permanente Center for Health Research) and Takeda Vaccines, Inc. (investigator-initiated research grants IISR-2015-101015 and IISR-2017-101938 to the Kaiser Permanente Center for Health Research). Takeda had no role in study design, data collection and analysis, decision to publish, or preparation of the manuscript. The CDC received no funding from Takeda.” |

*Give information separately for cases and controls in case-control studies and, if applicable, for exposed and unexposed groups in cohort and cross-sectional studies.

**Note:** An Explanation and Elaboration article discusses each checklist item and gives methodological background and published examples of transparent reporting. The STROBE checklist is best used in conjunction with this article (freely available on the Web sites of PLoS Medicine at http://www.plosmedicine.org/, Annals of Internal Medicine at http://www.annals.org/, and Epidemiology at http://www.epidem.com/). Information on the STROBE Initiative is available at www.strobe-statement.org.
